# Duty of care trumps utilitarianism in multi-professional obesity management decisions

**DOI:** 10.1177/09697330221075764

**Published:** 2022-05-27

**Authors:** Toni McAloon, Vivien Coates, Donna Fitzsimons

**Affiliations:** Department of Nursing, 42259 Ulster University - Jordanstown Campus, Newtownabbey, UK; Department of Nursing, 2596 Ulster University - Coleraine Campus, Coleraine, UK; Department of Nursing, 1596 Queen's University Belfast, Belfast, UK

**Keywords:** Obesity management, rights, equity, utility, multi-professional

## Abstract

**Background:**

Escalating levels of obesity place enormous and growing demands on Health care provision in the (U.K.) United Kingdom. Resources are limited with increasing and competing demands upon them. Ethical considerations underpin clinical decision making generally, but there is limited evidence regarding the relationship between these variables particularly in terms of treating individuals with obesity.

**Research aim:**

To investigate the views of National Health Service (NHS) clinicians on navigating the ethical challenges and decision making associated with obesity management in adults with chronic illness.

**Research design:**

A cross-sectional, multi-site survey distributed electronically.

**Participants:**

A consensus sample of nurses, doctors, dietitians and final year students in two NHS Trusts and two Universities.

**Ethical considerations:**

Ethical and governance approvals obtained from a National Ethics Committee (11NIR035), two universities and two teaching hospitals.

**Results:**

Of the total (*n* = 395) participants, the majority were nurses (48%), female (79%) and qualified clinicians (59%). Participants strongly considered the individual to have primary responsibility for a healthy weight and an obligation to attempt to maintain that healthy weight if they wish to access NHS care. Yet two thirds would not withhold treatment for patients with obesity.

**Discussion:**

While clinicians were clear about patient responsibility and obligations, the majority prioritised their duty of care and would not invoke a utilitarian approach to decision making. This may reflect awareness of obesity as a multi-faceted entity, with responsibility for support and management shared amongst society in general.

**Conclusions:**

The attitudes of this sample of clinicians complemented the concept of the health service as being built on a principle of community, with each treated according to their need. However limited resources challenge the concept of needs-based decisions consequently societal engagement is necessary to agree a pragmatic way forward.

## Introduction

Obesity levels worldwide are escalating with 39% (1.9 billion) of the adult population overweight and 13% (650 million) obese.^1^ The highest European obesity rates are evident in the UK with 63% of adults above a healthy weight, half of whom are obese.^2^ Obesity is associated with a range of co-morbidities such as type 2 diabetes mellitus (T2DM), hypertension, cardiovascular disease (CVD), liver disease and respiratory conditions.^3^ The adverse consequences of obesity, for the individual, public health and society in general, have been highlighted recently with the emergence of a new illness caused by the COVID-19 pandemic. Pandemic monitoring data indicates that obesity is associated with a more severe clinical course and increased risk of mortality, morbidity and requirement for invasive interventions.^4,5,6,7^ The causes of obesity are usually multi-faceted. While there may be contributing factors beyond an individual’s control, such as hormonal and genetic abnormalities, pharmacological side effects, psychiatric and socio‐cultural factors,^8,9^ its presence is also associated with individual lifestyle choices.^8^ Escalating obesity levels has placed growing demands on health care provision in the UK^3,10^ which is exacerbated by the COVID-19 pandemic and the demands on a health system reinstating normal services. As The National Health Service (NHS) is a publicly funded service resources are limited and how these resources are allocated raises ethical considerations that include how resources are most effectively used and equitably distributed. These are questions that are appropriate for an informed society to address, come to conclusions about, and make policy and funding decisions accordingly. In the absence of doing so, clinicians are left to make day-to-day decisions about treatment for patients with obesity in circumstances where the basis of decision making is not transparent.^11,12^ Furthermore, this also potentially results in *‘uncomfortable concern, anxiety, indecision or disputation over right actions’* for clinicians.^13^ Thus, this paper is focused on consideration of the moral tensions which may occur for clinicians when balancing the concepts of patient rights with public welfare in obesity management.

### Background

The right to self-determine features heavily in health care debates, but actions taken to manage the obesity pandemic potentially affect liberty and autonomy.^14^ It is reasonable to suggest that the foundations for autonomy in health care originated in Immanuel Kant’s principle of humanity^15^ and John Stuart Mill’s principle of liberty.^16^ Kant believed people have the capacity to be rational, set objectives, self-govern and therefore should self-determine. Mills asserted that if a decision is only related to the individual and does not have the potential to affect others, then the individual is at liberty to act regardless of the consequences. Conversely when others are at risk from an individual’s decision, it becomes subject to restrictions through deploying what Mills referred to as the ‘*harm principle*’, that is, others should be protected from harm caused by your decision. Respect for autonomy is seen as a priority obligation in health care services today, but it is a ‘prima facie’ obligation,^17^ meaning it can be overridden if there are other competing moral principles such as the just distribution of health care resources. Indeed, The Nuffield Council on Bioethics^18^ asserts that whilst all care should be taken to protect individual liberty, infringements are acceptable when the purpose is to protect others.

Protection of individual patient rights in health care maximises patient autonomy whereas protection of population rights maximises health outcomes for populations rather than individuals**.** The demands placed on the NHS by the obesity pandemic cannot be ignored, as there is a moral obligation for the NHS to behave justly, with equitable use of its limited resources to maximise outcomes.^10^ This has become even more evident post COVID-19 with pressures on the NHS to resume normal services and respond to the backlog of patients who remained untreated during the COVID-19 surges. Effective deployment of resources is challenging and often a utilitarian stance is taken, with the focus on maximising utility through trying to ensure the greatest benefit of the service for the greatest number of people. The application of utility can be controversial as it makes judgements based on population needs and the needs of the individual may be sacrificed in a move to increase overall benefit for a larger number of people.^14^ Maximising the use of resources in this manner conflicts with free choice by self-governing citizens and may trigger a moral tension for clinicians.^19^ Hence, moral tensions may be provoked by conflict between the libertarian theories of individual right to free choice and respect for autonomy and utilitarian theories of efficient use of U.K. scarce resources to benefit all.

The introduction and background literature are largely set within a UK perspective as this study was undertaken to examine the views of clinicians working in clinical NHS settings in which access to publicly funded health care is free at source which thus provokes additional finite resource considerations. Literature pertaining to other arenas of health care delivery such as private health care as practised in the United States of America (USA) was considered less relevant. Their inclusion would change the whole context of the discussion as those who can afford the service or who have sufficient insurance are not restricted by resource implications or a benefit/burden analysis. There is also a dearth of literature focusing on the ethical parameters of day-to-day multi-professional practice in obesity management with which to contextualise this paper. The existing literature is largely focused on specific issues such as funding bariatric or fertility services for those with obesity, not the daily challenges experienced by clinicians. Therefore, this paper through articulating the views of a large multi-professional sample helps bridge that gap in the literature.

### Research aim

To investigate the views of clinicians working in the NHS on navigating the ethical challenges that may occur when balancing the concepts of respect for an individual patient’s liberty with the utilitarian stance of maximising resources in managing patient obesity and chronic illness.

### Research questions

(1). Who do clinicians consider has primary responsibility for maintenance of a healthy body weight?
(2). Do clinicians consider that a right to access NHS health care includes an obligation to attempt a healthy lifestyle?
(3). In what circumstances do clinicians consider that treatment options should be limited for patients with obesity?

## Methodology

A cross-sectional, multi-professional multi-site survey was conducted electronically via a secure web-based platform administered by Project Implicit USA.

### Participants

To enhance participant anonymity access was gained via gatekeepers to clinicians that included qualified nurses, doctors, dietitians and final year students involved in the daily management of patients with obesity and chronic illness in two large NHS Trusts and two Universities in Northern Ireland. Gatekeeper credibility was crucial, as this was key to gaining the attention of potential participants. The gatekeepers varied by settings and included NHS Nursing and Dietetic Managers, Clinical Directorate Managers (Drs), Primary Care Practice managers, university Course Directors/Head of Divisions and university Heads of Schools. The study was promoted via the hospital intranet and by displaying posters/flyers.

### Ethical considerations

Ethical and governance approvals were obtained from a National Ethics Committee 11NIR035, two universities and two teaching hospitals in the U.K. and the study conformed to the Declaration of Helsinki.^20^ An introductory email containing a participant information pack with the study outline, rationale, participant requirements and a URL to enter the study site was sent via the gatekeepers to potential participants NHS or university email addresses. Participation was voluntary and anonymous with no identifiable data collected, consequently participation in the survey was considered to indicate consent. Participants could withdraw at any time and their anonymous electronic data was stored on a password protected computer.

### Measures

#### a) Demographic data

Demographic data comprised age, gender, experience, professional group and self-reported weight and height.

#### b) Survey items

Participants were asked to complete scales relating to four statements S1-S4 and to respond to one statement requiring qualitative commentary (S5). These survey items were generated after discussion with clinicians in the field and consideration of the available literature.^12,14^

### Statements

(1). Primary responsibility for maintaining a healthy weight rests with the individual (Likert scale *strongly agree 1 – strongly disagree 7).*
(2). The right to access NHS health care implies an individual obligation to attempt to maintain a healthy lifestyle *(Likert scale strongly agree 1 – strongly disagree 7).*
(3). The presence of significant obesity should restrict the available treatment options. *(1. Yes, 2. No).*
(4). The restriction of treatment options can be justified when (ranking of four given options generated from the literature base):
  (a). The patient does not agree to lose weight.
  (b). Non concordance with agreed weight loss plan.
  (c). Current level of obesity would significantly reduce the likelihood of successful treatment.
  (d). The obligation to maximise effective/efficient use of limited resources.
(5). Obesity is grounds for refusal of clinical treatment (example given of 15 Obstetric/gynaecological practices in the USA who declined to treat patients with obesity). Please add your comments to contribute to the discussion.

### Analysis

SPSS version 22 was used to provide summary statistics on the sample demographics. Participant responses were examined using descriptive and inferential statistics in accordance to the measurement type. For example: ANOVA was used to check if the means of the subgroups were significantly different to each other (doctors, nurses, dietetics and students) and interactive effects for nominal data were examined using 2-way ANOVA. Analysis of the qualitative data used content analysis to systematically transform the large amount of text into an organised and concise summary of results.^21^ Data were read to get a general feeling and understanding of what participants had stated. It was then re-read and divided up into smaller parts to gain meaningful units of information which were coded and labelled. At this stage, coding was descriptive, linked to the question posed which then led on to greater examination involving interpretation, sub-division of the text and modification of the codes. The transcripts were re-read to establish patterns which could be linked to become themes. Analysis of the data is presented under the emerging themes.

## Results

A multi-professional sample of clinicians (n = 395) provided demographic information. The majority had a nursing background, were predominately female and were qualified clinicians (see Table 1). A diversity of clinicians and clinical practice areas was represented, with clinicians from Coronary Care Units, Medical Wards, Outpatient Departments, Dietetic, General Practice and Community Practice areas. The mean calculated BMI for participants based on the self-reported weight and height measurements, was 25 kg/m^2^ (SD = 5.27). The distribution of BMI classification was 3% underweight, 58% normal weight, 26% preobese and 13% obese (classes i–iii). The combined classification of pre-obesity and obesity was highest in nursing (47%).

**Table 1.** The demographic characteristics of the sample.

Participant variable	n (%)
Gender (*n* = 392^a^)	
Male	83 (21)
Female	309 (79)
Age range (*n* = 395^a^)	
18–28	134 (34)
29–38	89 (23)
39–48	102 (26)
49–58	61 (15)
59–68	9 (2)
Professional group (*n* = 395^a^)	
Nurse	138 (35)
Nursing student	53 (13)
Doctor	69 (17)
Medical student	72 (18)
Dietitian	26 (7)
Dietetic student	7 (2)
Unknown	30 (8)
Clinical experience (*n* = 391^a^)	
Student	132 (34)
Qualified: <5 years	21 (6)
5–10 years	44 (11)
11–15 years	40 (10)
16–20 years	44 (11)
>20 years	110 (28)
BMI levels (*n* = 388^a^)	M = 25 kg/m^2^ (SD = 5.27)
Underweight (<18.50)	11 (3%)
Normal weight (18.50–24.99)	226 (58%)
Pre-obese (25–29.99)	101 (26%)
Obese class 1 (30–34.99)	38 (10%)
Obese class 11 (35–39.99)	4 (1%)
Obese class 111 (>40)	8 (2%)

Abbreviations: BMI: body mass index; M: mean; SD: standard deviation; n: number of participants.

^a^Some missing data.

The results reported below are aligned with respondents’ views on the concept of body weight, responsibility for a healthy weight and access to health care. Alongside the quantitative data qualitative data were collected from participants (*n* = 112) through responses to the open statement (S5). Some simply added yes/no (*n* = 21) responses but most added more detailed responses (*n* = 91). Analysis identified three main themes which were ‘*Rationale for body weight’*, *‘Patient rights and responsibilities’* and ‘*Justifications for treatment management’ *(Table 2). Analysis resulted in integration of the qualitative and quantitative data to illustrate the sample’s holistic views and is presented under the three identified themes.

**Table 2.** Qualitative Themes associated with obesity management.

Theme	Subtheme
1. Rationale for body weight	Underlying medical problem(s)
	As a comorbidity
	A pharmacological side effect
	Mental health problems/self-harm
	Personal lifestyle/personal control
	Relationship between food companies and governments
2. Patient rights and responsibilities	Equity if attempting to change weight
	Equitable treatment with other conditions
	Evidence of attempted weight loss needed
	Access to treatment requires taking responsibility
	Patient motivation to change needed
	Lack of fairness if obesity is unrelated to medical problem
3. Justification for treatment management	Not a HCP’s place to refuse treatment
	Hippocratic oath requirement
	Role of HCP to assist patients with weight loss
	Refusal of treatment an incentive to lose weight
	Compliance/lack of compliance
	Lifesaving versus elective treatment
	Risks versus benefits
	Relevance to health care problem
	Prospect for weight loss
	Potential for desirable consequences
	Obese at greater risk
	Weight loss for safety
	Treatment may not be possible
	Impact on resources
	Disheartening for clinicians

### Missing data

There were small amounts of random missing data relating to demographic characteristics and this was acknowledged in Table 1. The range for completion of data collection in respect of the four statements was 93–100%.

### Theme one *‘Rationale for body weight’*

Respondents’ consideration of the aetiology of obesity reflected contributing factors acknowledged in the literature base. It was recognised that the presence of contributing factors, including those beyond the individual’s control, made it difficult for individuals to exercise full governance over their behaviours and body status. This was illustrated by comments such as.

‘… (obesity)is self-inflicted but not for patients who suffer a clinical condition i.e., polycystic ovaries or depression’ (registered dietitian N4)’.

‘There are sometimes reasons that the person cannot fully be expected to be responsible e.g. medications can cause obesity’ (registered nurse N16).

‘… obesity is itself a medical problem which requires treatment in order to improve their quality of life and increase its longevity’ (registered dietitian N1).

Whilst a view was expressed that obesity could be a form of self-harm requiring psychological support it was also highlighted that environmental elements play a role.

‘I believe obesity is a type of self-harm issue. In my experience I have found obese people to have deep rooted psychological problems’ (registered nurse N6).

‘… the fundamental issues including food companies and the government relationship to them need to be tackled as part of overall plan’ (nurse specialist N1).

However, there was a thread running through all three themes which articulated that the development of obesity could be due to a personal lifestyle choice and if so, there may be consequences for the individual when accessing health care services.

‘…obesity is a lifestyle issue and unfortunately today’s world tends to facilitate this lifestyle. All the “nice things” are not always good’ (*nurse specialist N11*).

### Theme Two *‘Patient rights and responsibilities’*

When considering who held primary responsibility for maintaining a healthy weight 94% (*n* = 357) of respondents strongly agreed it was with the individual. The only variable which influenced this response was professional group (*p* = .01) in that student nurses held a slightly more neutral preference on whether the individual had primary responsibility for maintaining a healthy weight. Respondents elaborated on the concept of individual responsibility:

‘I feel if someone does not take responsibility for their health then they should not be given access to treatment where their obesity decreases the chance of success in the procedure’ (practice nurse N1).

‘I have personal experience with being obese and am now a healthy weight. It is possible. People will not change their weight until they finally accept that it is their responsibility to deal with it’ (nursing student N1).

If the individual wished to access NHS health care 72% (n = 286) of respondents articulated the individual was obligated to attempt to maintain a *‘healthy lifestyle’.* Participant BMI was the only significant demographic variable (*p* = .015) with this concept. Those participants on the upper end of obesity ranking, that is obese III, disagreed that an individual obligation to attempt to maintain a healthy lifestyle existed. Whilst almost three quarters of participants felt there was an obligation to maintain a healthy weight, they commented that when obesity was present those patients making positive attempts to manage their weight were viewed as deserving of encouragement and support as they were attempting to fulfil this obligation.

‘… depends on the reason for their obesity - if they show sufficient evidence that they have tried to lose weight then they should be considered for treatment’ (registered dietitian N8).

‘… patients should be encouraged to show evidence that they are trying to control their weight as part of their treatment. They should not be declined treatment’ (registered nurse N5).

Some clinicians considered that failure to moderate lifestyle choices would become a relevant consideration for subsequent access to health resources.

‘There is no need to overeat, it is all to do with portion size and exercise’ (registered dietitian N7).

‘… (withhold treatment) if the obesity is due to poor lifestyle choices and development of the disease in question akin to liver transplant to an alcoholic who refuses to abstain from alcohol,’ (medical student N7).

A number of respondents expressed views around equitable access to services. Comparisons were drawn with other conditions perceived to be linked to lifestyle choices. It was felt parity of access to health services should be given to those individuals with obesity as for those seeking access because of other lifestyle behaviours.

‘…is participation in sports grounds for not treating fractures?’ (GP N1).

‘… thin end of the wedge; why not refuse smokers or saturated fat eaters or those who take too little exercise or those who don’t live a perfect life?’ (consultant N8).

‘… if we refuse to treat patients where their behaviour has been a component in the development of their condition, we would treat virtually nobody’ (medical student N14).

### Theme Three ‘Justifications for treatment management’

**Figure 1.**
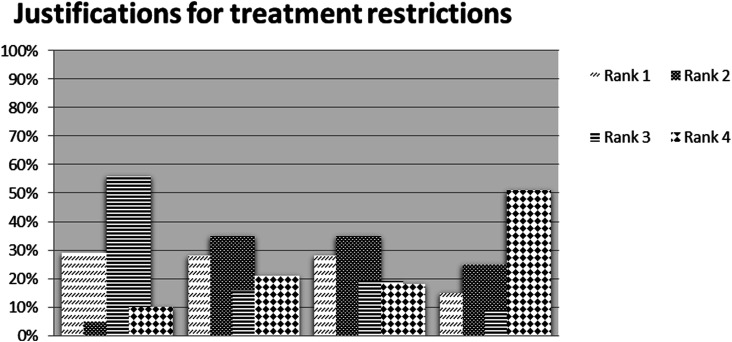
Respondent ranking of justifications for restriction of treatment options with patients with obesity.

Whilst the majority of respondents identified the individual as responsible for maintaining a healthy weight, 66% (*n* = 262) would not restrict treatment options in the presence of obesity. Professional group was the only demographic variable which significantly influenced the response with dietetic and medical students scoring more towards restricting treatment options than other professional groups (*p* = .005). Those individuals who supported treatment restrictions (*n* = 137) then ranked four provided justification options in order of 1–4 as their preference (Figure 1).

When considering the justification of treatment restrictions, the first option ‘*the patient does not agree to lose weight’* was ranked by 29% of participants as their number one justification, 5% as their second choice, 56% as their third choice and 10% as their fourth choice.

With option two ‘*non concordance with agreed weight loss plan’*, 28% ranked this as number one justification for treatment restrictions, 35% percent their second choice, 16% their third and 21% percent their fourth.

Option three the ‘*influence of the current level of obesity on successful outcomes’* was ranked as their first justification for treatment restrictions by 28% percent, second by 35%, third by 19% and fourth by 18%.

The final option regarding ‘*the obligation to maximise resources’* was ranked first choice by 15%, second by 25%, third by 9% percent and fourth by 51%.

Participant demographic variables were not statistically significant in influencing this ranking beyond professional group (*p* = .004) with nurses, student nurses and doctors favouring patient non concordance with agreed weight loss plan higher in their ranking of importance than other professional groups. Patient non agreement to lose weight was the dominant reason for restricting treatment and maximising use of limited resources least favoured. Indeed, consideration of restricting treatments provoked respondents to comment on their professional obligation of beneficence to their patients.

‘Hippocratic oath states to promise to do good for your patients it doesn’t specify that you can choose the patients’ (medical student N18).

‘… it is not any medical professional’s place to refuse medical treatment to any patient’ (registered nurse N21).

‘… refusal is unethical and reflects inequitable access to health care’ (GP N6).

Despite articulating justifications for treatment restrictions, some two thirds of our sample in practice would not restrict access to services. Indeed, there was an acknowledgement that patients with obesity are at a greater health risk and should not be denied treatment.

‘I strongly disagree that obesity should be grounds for refusal of treatment. Obese patients are more at health risks and so need readily available and accessible treatment’ (nurse specialist N7).

‘I think the person needs to be strongly encouraged with weight loss in order to help their treatment, however I do not think a person can be refused treatment based on their weight. Would you refuse to treat someone who is anorexic?’ (registered dietitian N5).

In the context of treatment, decisions made in daily practice assessments are undertaken routinely of risks versus benefits for the individual patient and treatment planned accordingly. Participants acknowledged that such evaluations would influence their decisions on the appropriateness of withholding treatment but would not present a situation for a complete ban.

‘(withhold treatment)… if it will pose an unacceptable risk due to the difficulties of surgery. Obviously if emergency/lifesaving treatment required then that is different and surgery should be done (if patient agrees) despite risks of obesity’ (consultant N3).

‘… it is grounds to limit the options on basis of risk. It cannot be a complete ban’ (specialist registrar 6 N1).

‘Only if their BMI constitutes a major risk of the treatment (e.g. surgery), and it is within their capability to change it (e.g. severe heart disease may require surgery but may limit capacity to lose weight)’ (consultant N7).

Respondents elaborated further on qualifiers to contextualise a decision to withhold treatment.

‘….depends on many factors - what is the treatment, lifesaving versus elective. Depends on degree of effort/motivation to address weight’ (consultant N1).

‘…only if the success of the treatment is negated by obesity – then it is futile exercise / waste of resources. If success of treatment does not depend on the patient’s weight then it becomes discrimination to withhold treatment’ (consultant N6).

Responses also indicated awareness of an obligation to utilise health resources effectively with some viewing that fairness to other patients also seeking access to limited resources is a valid factor when making decisions to allocate interventions.

(withhold treatment) ‘…not obesity on its own but where treatment is restricted and expensive, refusal to comply with advice on weight management, particularly if there are other more cooperative patients on the waiting list’ (medical student N6).

*‘…if they are continuously relenting to change and other people need treatment I can understand refusal’* (nursing student N15).

## Discussion

The overwhelming professional consensus from our novel study was that primary responsibility for a healthy weight rests with the individual (95%) and that there was an associated obligation to at least attempt to maintain a healthy lifestyle (72%). Similar views were expressed by the general public in the U.K. when IPSOS Mori recently conducted a survey for the Health Foundation^22^ with 97% of respondents citing the individual as having a ‘*great deal’* to a ‘*fair amount’* of responsibility for generally staying healthy. These combined findings suggest that there might be a common view in society that, in matters of health care provision, respect for individual autonomy is qualified and there are circumstances where competing ethical principles could take precedence.^18^ This position would reflect Mill’s ‘*harm principle’*^16^ view of autonomy, in that the individual only has the right to have ‘*liberty*’ in decision making if there is no harm caused to others. In the context of obesity, potential harm to others could include not maximising effective use of a society’s finite health care resource. There is however the potential for other harms to arise if this is the only harm taken into consideration. The new government obesity strategy^10^ has been criticised as endorsing guilt and shame for those with obesity because they are asked to lose weight to ‘*reduce pressure on doctors and nurses in the NHS, and free up their time to treat other sick and vulnerable patients*’ (pg4). Moreover, targeting the individual for blame has been criticised as an ineffective public health strategy.^23^

While our clinicians were clear in their views on patient responsibility and obligation the majority, two thirds, would not adopt a utilitarian approach when it comes to decisions on the right to access health care and the allocation of resources. This finding may reflect acknowledgement that the aetiology of obesity can be multi-faceted and conceptualised as not merely an individual failing but also as a failure of the environment and the state to support the individual with regard to healthy behaviour.^19,24,25^ NHS support for healthy behaviour is also reflected in Mori public polling^22^ which reported a majority view that the government and the NHS has either a ‘*great deal’* or ‘*fair amount’* of responsibility for ensuring people generally stay healthy. The majority of our clinicians’ views though are at odds with the position of some NHS Clinical Commissioning Groups (CCG) who have applied mandatory rationing measures when seeking access to surgery for certain groups. For example, The Vale of York CCG’s policy requires patients with a BMI greater than 30 to lose 10% of their body weight or sustain a 12-month period of attempted weight loss prior to being referred for elective surgery.^26^ In a Royal College of Surgeons (RCS) review of CCG thresholds, 39% of respondents said their CCG was considering new limits on eligibility for services, based on a utilitarian calculation of financial value and efficiency.^27^ It is possible that health care professionals modify their views depending on whether the hat being worn at the time is clinical or managerial. It could also be the case that clinicians who selfselect to be involved in overt decision making about resource allocation are more comfortable with the utilitarian approach to start with and vice versa. However, if the proposition that the key to success with obesity management is actually promoting shared responsibility between the individual and the state^28,29^ is correct, our participants’ reluctance to restrict access to NHS services would support a strategy of encouragement of positive autonomous health related behaviours rather than adopting what could be viewed as a punitive one. Thus, future development necessitates clinicians working closely with communities to ensure that all members of the community understand the principles that this shared responsibility are based on, agree with them and consequently act to rely on scarce resources as little as possible. Additionally, such real time examples as our study, illustrating the complexities of navigating ethically challenging clinical scenarios and the resultant moral tensions felt by clinicians, will be of value to those involved in teaching healthcare ethics.

Some difference in views was noted in participants with obesity and in those who were student professionals when compared to the rest of the sample. Participants with obesity agreed less with the concept of personal responsibility for maintaining a healthy weight. This study does not provide a reason for this. It is known that people with obesity are often stigmatised as lazy, weak willed and risk takers^30,31^ and it is possible that this characterisation influenced some responses. It is also possible that personal obesity is a potential barrier to clinicians advocating for taking personal responsibility for healthy weight maintenance and are thus less likely to expect it from patients. Student nurses tended to be neutral on this issue. This ambivalence may have reflected a combination of their limited personal experience and the finding that nursing had a higher prevalence of obesity than was the case in the other professional groups. Of the minority of clinicians that would limit resources, lack of agreement by the patient to lose weight was the commonest reason identified and the goal of maximising use of resources in terms of effectiveness and efficiency was ranked last. Dietetic and medical students were more likely to restrict available treatment options in the presence of significant obesity. A possible explanation for this is that inexperience and a novice appreciation of the complexities of clinical practice may influence conclusions reached.

The thematic analysis of the free text provided by those who addressed the issue of refusal of medical treatment indicated not only insightful awareness of the range of considerations involved, but also a sense that the respondents were probably experienced clinicians who understood the potential patient journey and articulated their primary duty of care. This element was captured in the comments relating to gauging beneficence for the patient through assessment of risks and burdens of treatment and the professional obligation for using health resources effectively and fairly. Consideration of fairness was multi-faceted as both the need for fair access to services for all and consistency when treating perceived ‘*self-inflicted’* conditions was acknowledged.

Potential limitations of this study include the use of an opportunistic rather than a random sample of clinicians and the dependence on an internet mediated survey, rather than face-to-face encounters which could enable greater exploration of responses. However, both these approaches were considered justified as the subject area was a sensitive issue and a guarantee of anonymity was more likely to gain honest responses and decrease social desirability influenced responses. Furthermore, whilst bias is possible in our study the sample represented both genders, all age groups, experience and BMI levels and the qualitative data collected demonstrated such varied responses that we were not led to believe it was a biased sample. As there is a dearth of published literature in this field future research would benefit from face-to-face interviews with multi-professional samples to inform clinical decision making through a deeper exploration of the concepts articulated by our sample.

## Conclusions

This study aimed to investigate the views of NHS clinicians on the ethical challenges experienced when balancing respect for individual liberty with maximisation of resources in managing patients with obesity**.** Our multi-professional sample expressed majority views consistent with the perspective of the Nuffield Council on Bioethics^18^ that considers the U.K. health service as built on a principle of community, in which each is treated according to need, with individuals treated as ‘*moral equals worthy of respect’*.^25^ This principle does however incorporate assumptions. Firstly, that all societal members understand the community principles approach, secondly are in agreement and finally will rely on scarce resources as little as possible. In the context of obesity this creates an obligation to attempt to maintain a healthy body weight which our sample endorsed. Obesity can be considered however, not as a personal failure, but rather as a result of multidimensional inadequacies, including a failure of the environment to promote and of the State to support individuals in maintaining healthy behaviour.^19,24,25^ The serious impact of the recent COVID-19 pandemic on availability of NHS provision, has served to highlight acutely the lack of a robust method for society to be involved with clinicians meaningfully in transparent decision making regarding resource utilisation and allocation in general, and for obesity management in particular. Such societal engagement needs to take place to reach an agreed pragmatic way forward and the insights gained in this study can be built upon to support transparent decision making.
